# ESG and corporate performance: The moderating role of government subsidies and mediating effect of analyst coverage in Chinese A-share listed companies

**DOI:** 10.1371/journal.pone.0322190

**Published:** 2025-05-08

**Authors:** Chuqiao Tang, Xiaolong Guo, Xuan Li

**Affiliations:** 1 Jinan Birmingham Joint Institute, Jinan University, Guangzhou, Guangdong, China; 2 Department of Earth and Environmental Sciences, St. Francis Xavier University, Antigonish, Nova Scotia, Canada; Southwestern University of Finance and Economics, CHINA

## Abstract

In recent years, the heightened emphasis on sustainable and high-quality economic development has garnered substantial investor interest in corporate ESG performance, significantly influencing the long-term operational stability of firms. This study, based on data from A-share listed companies from 2015 to 2022, explores the relationship between corporate ESG performance and corporate financial performance. This research employed regression analysis to examine this relationship and found that improved ESG performance significantly enhances financial performance, especially in state-owned enterprises and the manufacturing sector. Additional analysis shows that government subsidies positively influence the relationship between ESG performance and corporate financial performance, suggesting that subsidies amplify the positive effects of ESG initiatives on performance. Mechanism tests suggest that increased analyst coverage is a key pathway through which ESG performance boosts corporate financial performance. These findings underscore the importance of ESG initiatives for companies and provide empirical evidence supporting the role of government subsidies and analyst coverage in amplifying the positive impact of ESG performance on financial outcomes.

## Introduction

In recent years, social issues associated with sustainable development, including climate change and public health crises, have garnered significant global attention. China, as the world’s second-largest economy, has increasingly emphasized sustainable development, integrating the philosophy that “lucid waters and lush mountains are invaluable assets” [1] and announcing goals to peak carbon dioxide emissions by 2030 and achieve carbon neutrality by 2060. Recent studies demonstrate how green financial policies and institutional investors jointly drive ESG development in Chinese markets, improving access to green financing and reducing capital costs [2,3]. China’s unique market characteristics influence both its economic and environmental policies [4], placing it in a pivotal position to advance global sustainability goals [5]. As ESG becomes a crucial metric for sustainable development, governments have strengthened supervision of corporate ESG performance through various policies and regulations. Despite China’s burgeoning role in sustainability initiatives, the existing literature on ESG disclosure in this market remains limited, particularly regarding its unique institutional context. Therefore, it is imperative to examine how ESG practices manifest in China’s distinct regulatory environment.

China has implemented comprehensive policies to promote sustainability and transparency through ESG disclosure requirements. Key regulations include the Shanghai Stock Exchange’s “Science and Technology Innovation Board Stock Listing Rules” [6] and the “Green Finance Regulations of Shenzhen Special Economic Zones” [7], which mandate ESG information disclosure. In alignment with the well-established ESG frameworks in the U.S. and Europe, China has developed its own guidance system through documents such as the “Guidelines for Establishing the Green Financial System” and the “Measures for the Administration of Enterprise Environmental Information Disclosure” [3,8–11]. However, compared to the U.S. and EU markets, China’s ESG legislative framework is distinct, particularly in its requirements based on company type and size [12–15]. Listed companies and large enterprises must comply with rigorous mandatory disclosure standards covering environmental impact, social responsibility, governance practices, and long-term sustainability goals [15,16], while SMEs are encouraged to provide simplified voluntary disclosures [14,17]. This tiered regulatory structure has reinforced ESG as a crucial criterion for investment and consumption decisions [18]. An increasing number of investors and consumers are focusing on corporate ESG performance, driving companies to enhance their ESG management levels not only for policy compliance but also to maintain competitiveness [16]. Understanding China’s unique ESG framework provides valuable insights into how emerging markets might shape global ESG trends, offering implications for other emerging economies.

Existing literature has explored the ESG-performance relationship from three main perspectives. First, from an operational perspective, ESG performance enhances corporate development through multiple channels, including improved green innovation quality [19], increased corporate performance [20], investment efficiency [21], and enhanced risk management capabilities [22,23], with recent methodological advances providing more precise measurements through new evaluation frameworks [24] and machine learning techniques [25]. Second, from a market perspective, strong ESG performance influences various stakeholders - for financial intermediaries, it attracts greater analyst coverage [26] and institutional ownership [27], with improved ESG disclosure quality leading to 30% higher analyst coverage [28] and more accurate analyst forecasts for firms with better ESG performance [29], while for capital markets, it eases financing constraints [30], reduces default risks [22], and lowers stock price crash risks [23]. The government’s role has become increasingly significant, with studies documenting how subsidies strengthen ESG implementation [31] and improve environmental performance [32]. However, the relationship between ESG and financial performance remains debated, with competing views emerging: the positive view suggests that ESG enhances financial outcomes through reduced information asymmetry [33], increased media attention [26], improved corporate performance [34], and enhanced corporate efficiency through easing financing constraints [35]; the negative view argues that ESG initiatives increase costs and harm financial performance [36]; while a neutral perspective finds no significant relationship [37]. Studies in the Chinese context reveal more complex patterns, including threshold effects [38], negative correlations [39], and temporal variations where ESG exhibits a substitution effect in the short term but transitions to a promotional effect in the long term [40], while Yu and Xiao [41] find a positive correlation. These conflicting findings can be attributed to several factors: methodological differences in ESG measurement lead to divergent results [28,42], institutional contexts significantly affect outcomes between developed and emerging markets [43], and the mechanisms linking ESG to performance are potentially non-linear and context-dependent [44]. Several important gaps remain unexplored: (1) the role of China’s unique institutional environment, (2) variations across industries and ownership types in ESG impact, and (3) the influence of external factors such as government policies and market supervision mechanisms. Therefore, this study aims to fill in these research gaps and further reveal the internal relationship and the influencing factors behind it through in-depth analysis of the data of A-share listed companies. Clarifying this issue can help promote the enhancement of corporate performance and support coordinated and sustainable economic and social development.

Based on the review of existing literature and current research gaps, three critical questions emerge that warrant investigation. First, while studies have documented various relationships between ESG and firm performance, how this relationship manifests in China’s unique institutional context remains unclear. Second, given China’s strong government influence in economic development, how do government subsidies affect the ESG-performance relationship? This question is particularly relevant as recent policies emphasize both ESG development and efficient resource allocation. Third, in China’s evolving capital market where information asymmetry remains high, what role do market intermediaries, specifically analysts, play in translating ESG performance into firm value? To address these questions, this study develops an integrated framework examining both the direct and indirect pathways through which ESG performance affects firm value. This framework considers the moderating role of government subsidies and the mediating effect of analyst coverage, reflecting the unique institutional characteristics of China’s market. By simultaneously examining these mechanisms, we can better understand how institutional support and market intermediaries jointly influence the effectiveness of ESG initiatives.

This paper examines the impact of corporate ESG performance on financial outcomes and explores the underlying theoretical mechanisms. Based on this, it utilizes data from Tobin’s Q and the China Securities Index (CSI) ESG ratings of A-share listed companies from 2015 to 2022 to analyze the relationship between corporate ESG performance and financial performance. Furthermore, this paper examines potential influencing mechanisms from the perspectives of government subsidies and analyst coverage. The empirical results indicate that ESG performance boosts corporate performance, with stronger effects in state-owned enterprises due to their stricter regulatory oversight, and in the manufacturing sector given its significant environmental impact. Additionally, ESG performance can improve corporate performance by increasing analyst coverage. This study aims to provide firms and investors with empirical evidence on the importance of ESG initiatives, while revealing the role of government policies and market surveillance mechanisms in amplifying the positive impact of ESG performance on firms’ financial performance.

The incremental contributions of this study are as follows: First, while previous studies have primarily focused on direct ESG-performance relationships, this study extends recent literature by examining the complex interactions between ESG initiatives, government support, and firm performance. Our findings provide new evidence on how policy interventions can amplify ESG effectiveness, responding to calls from recent studies for more nuanced understanding of policy-performance relationships. Second, existing research on ESG performance typically focuses on internal mechanisms such as debt financing costs and financial risks. This study advances the literature by examining external transmission channels, particularly the role of analysts as information intermediaries. Our analysis builds on emerging work by Zhang and Wu [26] about market participants in ESG value creation, offering new insights into how analyst coverage mediates ESG impacts. Third, we contribute to recent discussions on ESG heterogeneity by documenting significant variations across ownership structures and industries, showing how institutional and sector-specific factors shape ESG effectiveness. Finally, by integrating these multiple perspectives, our study provides a more comprehensive framework for understanding ESG value creation in China’s unique institutional context, offering practical implications for both policy makers and corporate managers.

## Research hypothesis

### ESG performance and corporate outcomes

The relationship between ESG performance and corporate outcomes is effectively explained by stakeholder theory and signaling theory. Stakeholders are intimately linked to the company, and their actions can profoundly influence corporate performance [45,46]. Firms must thus ensure transparency in disclosing corporate data to mitigate information asymmetry and enhance investor trust [47]. By fulfilling environmental responsibilities, companies send positive signals to external stakeholders, which helps in gaining external support and trust. Financial institutions offer low-cost financing and issue green bonds to companies with strong ESG performance, thereby reducing financial expenses and enhancing profitability. Investors are likely to increase their shareholding and provide financial support to companies with good ESG performance, offering more comprehensive guarantees for profitability. Stakeholders also help companies improve their intangible assets [48,49], optimize capital expenditures [50], and investments [51], thereby enhancing corporate performance.

Resource dependence theory also provides a reasonable explanation for the link between ESG performance and corporate performance. The theory posits that for companies to establish and grow in the market, they need not only their own strength but also external resources. Good ESG performance demonstrates a company’s proactive approach to social responsibility, helping it to secure crucial strategic resources [52], enhance competitive advantage [53], boost investor and consumer trust [54], and solidify business partnerships [55]. This can lead to expanded production and operation scales, thereby improving corporate efficiency.

Furthermore, good ESG performance indicates that a company actively fulfills its obligations, significantly reducing its risks in environmental, social, and governance areas, thereby lowering operational risks. This helps to regulate corporate behavior and promote performance improvement. By effectively managing resources and reducing waste, businesses can attain both sustainability and financial success [56]. Most of the previous research has demonstrated that ESG performance has a positive impact on the improvement of enterprise performance [57–59]. Based on the above analysis, this paper presents the following hypothesis:

**H1.** Corporate ESG performance has a positive impact on corporate performance.

### The moderating role of government subsidies

Government subsidies, as an incentive measure provided by the government to enterprises, promote corporate development through tax reductions, R&D funding, green compensation, and other means [60]. The moderating effect of government subsidies on the ESG-performance relationship can be understood through multiple theoretical frameworks. From a resource dependence perspective, government subsidies provide firms with critical resources that enhance their capacity to effectively implement ESG initiatives [61]. This resource support enables firms to make more substantial investments in environmental protection, social responsibility, and governance improvements, thereby strengthening the positive impact of ESG activities on performance. Institutional theory further suggests that government subsidies signal strong institutional support for ESG practices. By providing subsidies, the government creates both incentives and expectations for improved ESG performance [60]. This institutional alignment motivates firms to maintain high ESG standards to preserve their legitimacy and continue receiving government support.

Through these mechanisms, government subsidies can amplify the ESG-performance relationship in several ways. First, subsidized firms have greater financial flexibility to invest in ESG initiatives and increase R&D investment [62], potentially leading to more effective implementation and innovation [63]. Second, the institutional recognition represented by government subsidies reduces information asymmetry in the market and creates a “certification effect” that enhances investor trust and attracts more social capital [64,65]. Third, to maintain their preferred status and continue receiving subsidies, firms are motivated to ensure their ESG investments translate effectively into performance improvements [60]. Evidence from emerging markets supports these theoretical arguments, particularly in contexts where government support plays a significant role in corporate development. Companies receiving government subsidies demonstrate enhanced capacity to translate ESG initiatives into improved operational efficiency [66], innovation outcomes [63], and overall financial performance [67]. Recent research by Zhu et al. [68] further confirms that government subsidies have a significant positive moderating effect on the relationship between enterprise ESG and performance improvement. Based on the above analysis, this paper proposes the following hypothesis:

**H2.** Government subsidies have a positive moderating effect on the impact of corporate ESG performance on corporate performance.

### ESG performance, analyst coverage, and corporate performance

The mediating role of analyst coverage in the ESG-performance relationship can be understood through information intermediary theory and monitoring theory. Information intermediary theory suggests that analysts serve as sophisticated information processors in capital markets [69]. Through their professional expertise and industry knowledge, analysts effectively translate complex corporate information into market signals, helping reduce information asymmetry between firms and investors [70]. The monitoring theory posits that analysts, as external monitors, play a crucial oversight role that can enhance corporate governance and operational efficiency [71].

Strong ESG performance signals a firm’s commitment to sustainable development and social responsibility [72], which attracts analyst attention through multiple channels. First, ESG initiatives provide additional non-financial information that analysts can use to evaluate firm value more comprehensively [73]. Second, strong ESG performance often indicates better risk management and long-term strategic planning, aspects that analysts value in their assessments [74]. The relationship between ESG performance and analyst coverage may exhibit non-linear patterns, as the marginal impact of ESG improvements on analyst attention could vary at different performance levels [38].

The mediating effect of analyst coverage on firm performance operates through dual mechanisms. As information intermediaries, analysts help market participants better understand and value firms’ ESG initiatives, potentially leading to improved market recognition and financing opportunities [27]. Their monitoring role encourages firms to maintain high ESG standards and operate more efficiently. Evidence from emerging markets suggests that analyst coverage is particularly valuable in contexts where information asymmetry is high. This effect becomes especially pronounced in markets like China, where analysts play a crucial role in validating and disseminating ESG-related information to market participants [26]. Based on this analysis, this paper proposes the following hypothesis:

**H3.** Corporate ESG performance can enhance its performance by increasing analyst coverage.

**Fig 1:**
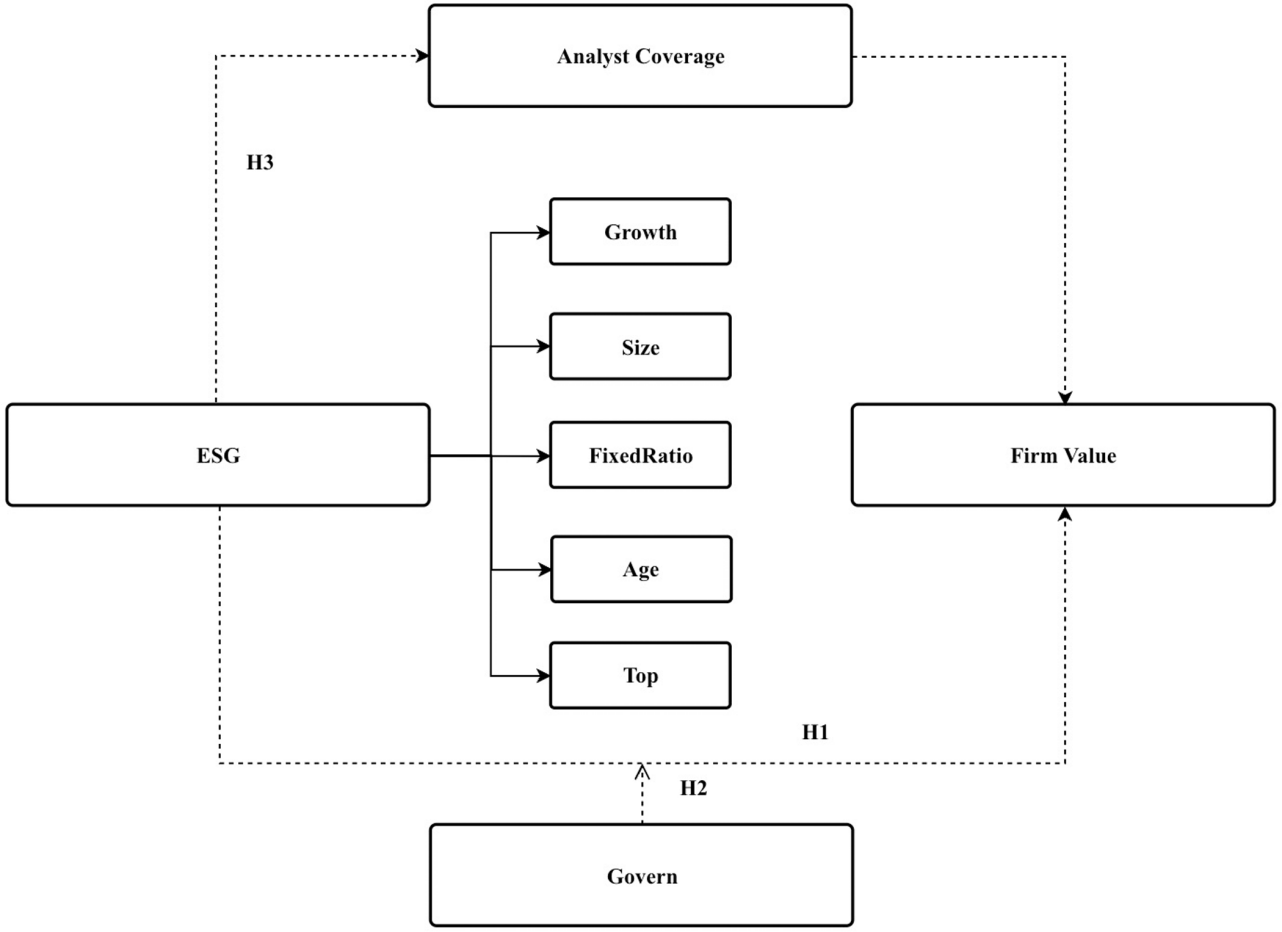
The influence mechanism of ESG performance on firm performance through analyst coverage and governance.

This paper establishes a mechanism framework based on logical reasoning to illustrate how ESG performance impacts enterprise value. The framework comprises three elements: analyst coverage [26,27], governance, and firm value [27], as depicted in Fig 1.

## Research design

### Data sources and sample selection

In order to verify the above hypotheses, this paper selects A-share listed companies from 2015–2022 as the research sample, and in order to ensure the accuracy of the data, this paper excludes the following data: (1) Companies with special financial status, such as ST and *ST are excluded. These companies often face financial difficulties or other significant risks, and their financial data and business performance may differ significantly from those of other companies. (2) Financial industry companies are excluded due to their unique business models and financial structures, which are not representative of other industries. (3) Companies with missing data for key variables, such as ESG performance or financial indicators, are excluded to ensure the completeness and reliability of the analysis. For companies with only one year’s data, this paper excludes them. Ultimately this research obtains a total of 5,666 firm-year observations. Among them, the ESG rating data come from the China Securities Index (CSI) ESG rating indicators in the Wind database, and the rest of the financial indicators and corporate governance data come from the CSMAR database. In this paper, the continuous variables are shrink-tailed at the upper and lower 1% levels to avoid the impact of outliers on the empirical results.

### Selection of variables

#### Explanatory variable: Firms’ ESG performance (ESG).

The selection of CSI ESG ratings as the primary measure is based on several key considerations. Compared to international ESG rating providers such as MSCI and FTSE Russell, CSI ratings are specifically designed for the Chinese market, incorporating local regulatory requirements and market characteristics [75]. Additionally, CSI’s rating methodology follows a systematic and comprehensive framework that evaluates multiple dimensions of corporate sustainability, making it particularly suitable for the Chinese context [76]. Furthermore, the CSI database provides more complete coverage of A-share listed companies, minimizing potential selection bias that might arise from using international rating systems with limited coverage of Chinese firms [77]. The CSI ESG rating system employs a comprehensive evaluation framework incorporating environmental (E), social (S), and governance (G) dimensions [78]. This framework consists of 13 major themes and 22 evaluation units covering approximately 200 indicators. The environmental dimension evaluates aspects such as climate change, pollution management, and natural resource utilization. The social dimension focuses on stakeholder relationships, including employee relations, supply chain management, and customer engagement. The governance dimension assesses shareholder rights, corporate structure, and operational management. The CSI ESG rating indicators are 9 grades from low to high, C, CC, CCC, B, BB, BBB, A, AA, AAA, and assigning them in order from 1 to 9. However, business-related social, environmental, and governance matters are interconnected, so focusing solely on one aspect does not provide a comprehensive view. Therefore, to provide a more nuanced understanding of sustainability impacts, this research examines the effects of individual environmental (E), social (S), and governance (G) components on corporate performance additionally.

#### Explained variable: Firm performance (TobinQ).

Following the methodology of Wong et al. [79], Ghosh et al. [80], Pareek and Sahu [81], Misani and Pogutz [82] this paper employs Tobin’s Q to measure corporate performance. Compared with other indicators, Tobin’s Q is more suitable for measuring enterprise performance [83,84]. Tobin’s Q is calculated by dividing the sum of market capitalization, total liabilities, preferred equity, and minority interest by total assets.

#### Mediator variable: Analyst coverage (Analyst).

Following the methodology of Chen et al. [85], this paper uses the natural logarithm of the number of analysts following a company, plus one, as the measure of analyst coverage.

#### Moderating variable: Government subsidies (Govern).

Referring to the research by Zhang and Xu [86], this paper uses the natural logarithm of the total government subsidies received by the company in the current period as the measure of government subsidies.

#### Control variables (Control).

This paper controls for the factors that explain firm performance, while isolating the pure effects associated with ESG performance. The selection of control variables is based on established theoretical frameworks and prior empirical evidence. Firm size (*Size*) is included as it affects organizational efficiency and market power, where larger firms may benefit from economies of scale but might face increased organizational complexity [87]. Financial stability (*FixedRatio*), measured as the ratio of fixed assets to total assets, reflects a firm’s operational leverage and risk profile in different market conditions [88]. Ownership Concentration (*TOP*), measured by the shareholding ratio of the largest shareholder, captures ownership concentration effects, which is particularly relevant in the Chinese market context where ownership structure significantly influences corporate decision-making [89]. Firm age (*Age*) controls for life-cycle effects, as mature firms may benefit from established market positions and operational experience but might face challenges in maintaining growth [90]. Growth potential (*Growth*), measured by revenue growth rate, captures a firm’s developmental stage and market opportunities, which significantly influence firm valuation and performance expectations in capital markets [91]. These variables collectively account for firm-specific characteristics that could affect the relationship between ESG performance and corporate outcomes. Table 1 provides the descriptions and specific measurement methods of the main variables.

**Table 1:** Variable definitions.

Variable Type	Variable Name	Variable Symbol	Variable Measurement
Dependent	Tobin’s Q	*TurbinQ*	(Market value of circulating shares + number of non-circulating shares × net assets per share + book value of liabilities)/ total assets
Independent	Firm ESG Performance	*ESG*	China Securities ESG rating
Mediating	Analyst Coverage	*Analyst*	Natural log of the number of analysts tracking the firm plus one
Moderating	Government Subsidy	*Govern*	Natural log of the total amount of government subsidies received by the firm during the current period
Control	Firm Size	*Size*	Natural log of the company’s total assets
	Ratio of Fixed Assets	*FixedRatio*	Fixed assets/ total assets
	Shareholding Ratio of Top Shareholder	*Top*	Share count held by the principal shareholder/ total shares
	Firm Age	*Age*	Natural log of (current year - year of establishment + 1)
	Revenue Growth Rate	*Growth*	Revenue growth/ revenue of the previous year

### Model specification

To verify the impact of ESG performance on corporate performance, the following model is constructed in this paper:

${TobinQ}_{i,t}\;=\;\beta_{0}+\beta_{1}{ESG}_{i,t\;}+{\;\beta }_{2}{Control}_{i,t}+{Industry}_{i}+{Year}_{t}\;+\varepsilon_{i,t}$ (1)

where ${TobinQ}_{i,t}$ represents the performance of firm i in year t, ${ESG}_{i,t}$ is the ESG rating of firm i in year t, ${Control}_{i,t}$ represents control variables, and $\varepsilon_{i,t}$ is the random disturbance term. Model (1) controls for industry (${Industry}_{i}$) and time (${Year}_{t}$) effects. When $\beta_{1}$ is greater than 0, it indicates that better ESG performance leads to better corporate performance.

## Empirical results and analysis

### Descriptive statistics

Descriptive statistics for the main variables are presented in Table 2. The maximum value of Tobin’s Q (*TurbinQ*) is 9.87, and the minimum value is 0.825, indicating a significant performance variation among the sample firms. The maximum value of ESG performance (*ESG*) is 8, with an average of only 4.15, suggesting that the overall ESG performance of the sample firms is relatively low, with considerable room for improvement. The maximum value of government subsidies (*Govern*) is 20.75, while the minimum is only 10.55, indicating a large disparity in government subsidies received by the sample firms. The mean value of analyst coverage (*Analyst*) is 2.004, indicating that listed companies in China are relatively easily covered by analysts. The maximum value of the largest shareholder’s ownership ratio (*Top*) is 70.53, with an average of 32.45, indicating a high concentration of ownership in some firms in China.

**Table 2:** Descriptive statistics.

Variable	N	Mean	Std. Dev	Min	Max
*TurbinQ*	5,666	2.454	1.687	0.825	9.870
*ESG*	5,666	4.150	1.080	1	8
*Top*	5,666	32.45	14.13	8.770	70.53
*FixedRatio*	5,666	0.215	0.152	0.00227	0.664
*Size*	5,666	22.72	1.156	20.43	26.21
*Age*	5,666	2.994	0.283	2.197	3.584
*Growth*	5,666	0.191	0.399	-0.470	2.602
*Govern*	5,666	16.59	1.836	10.55	20.75
*Analyst*	5,666	2.004	0.911	0.693	3.850
**VIF test**					
**Variable**			**VIF**		
*ESG*			1.07		
*Top*			1.03		
*FixedRatio*			1.03		
*Size*			1.49		
*Age*			1.02		
*Growth*			1.02		
*Govern*			1.28		
*Analyst*			1.23		

The research uses variance inflation factor (VIF) to test multicollinearity. The results show that the VIF statistic with Tobin’s Q as the dependent variable ranges from 1.02 to 1.49. The results are all less than 10 [92], which indicates that there is no serious multicollinearity problem between exogenous variables.

### Analysis of benchmark regression results

Table 3 reports the regression results of the impact of corporate ESG performance on performance. To avoid the impact of clustering effects on standard errors, clustered robust standard errors were used in the regression. Column (1) does not control for industry and year fixed effects, showing that the regression coefficient of ESG performance (*ESG*) on corporate performance (*TurbinQ*) is 0.091, significant at the 1% level. Column (2) controls for industry and year fixed effects, showing a regression coefficient of 0.114 for ESG performance (*ESG*) on corporate performance (*TurbinQ*), also significant at the 1% level. Considering environmental, social and governance impact, the result shows that social and governance performance have a significant positive impact on corporate performance, with coefficients 0.108 and 0.130 respectively, significant at the 1% level. These results indicate that better ESG performance can significantly enhance corporate performance, supporting hypothesis H1.

For the control variables, firm size (*Size*) has a negative impact on corporate performance, showing a regression coefficient of -0.556, significant at the 1% level. It suggests that as firms grow larger, issues such as employee redundancy and coordination difficulties may arise, leading to reduced performance. The largest shareholder’s ownership ratio (*Top*) has a positive impact on corporate performance, showing a regression coefficient of 0.009, also significant at the 1% level. It indicates that higher ownership concentration may facilitate better corporate governance and performance improvement. Revenue growth rate (*Growth*) also positively impacts corporate performance, with a regression coefficient of 0.513, significant at the 1% level. The result is consistent with the phenomenon that higher growth rates are associated with better corporate performance.

### Robustness tests

To ensure the robustness of the regression results, the following robustness tests were conducted in this paper.

#### Changing the dependent variable measurement.

Return on assets (*ROA*) is used as the dependent variable. Following the approach of Fujii et al. and Lestari et al. [93,94], *ROA*, which reflects a firm’s profitability, is used to represent corporate performance. The regression results (Table 4) indicate that the coefficient of ESG is 0.01 at the 1% significance level, showing that ESG is still significantly positively correlated with corporate performance, indicating that the results of this paper are robust.

**Table 4:** Robustness test results.

Variable	(1)	(2)	(3)
	*ROA*	*TurbinQ*	*TurbinQ*
*ESG*	0.010^***^		
	(8.45)		
*ESG_r*		0.177^***^	
		(3.17)	
*ESG_d*			0.111^***^
			(4.25)
*FixedRatio*	-0.041^***^	-1.622^***^	-1.573^***^
	(-4.53)	(-7.25)	(-7.34)
*Size*	-0.003^**^	-0.545^***^	-0.596^***^
	(-2.46)	(-16.01)	(-18.58)
*Age*	0.003	-0.135	-0.133
	(0.60)	(-1.00)	(-1.04)
*Top*	0.001^***^	0.009^***^	0.010^***^
	(5.72)	(3.82)	(4.20)
*Growth*	0.054^***^	0.507^***^	0.505^***^
	(11.20)	(8.24)	(8.02)
*Constant*	0.051	14.874^***^	15.873^***^
	(1.58)	(18.82)	(21.23)
Industry	Yes	Yes	Yes
Year	Yes	Yes	Yes
Observations	5,666	5,666	5,066
Adj R^2^	0.141	0.340	0.378

Note: t-values are shown in parentheses, and results are firm-level clustered;

* ,

** , and

*** indicate significance at the 10%, 5%, and 1% levels, respectively.

#### Changing the independent variable measurement.

Based on the CSI ESG ratings, ratings of C, CC, and CCC are assigned a value of 1; ratings of B, BB, and BBB are assigned a value of 2; and ratings of A, AA, and AAA are assigned a value of 3. Using *ESG_r* as the independent variable, the regression results (Table 4) show that the coefficient of *ESG_r* is 0.177 at the 1% significance level, indicating that ESG is significantly positively correlated with corporate performance, consistent with the conclusions drawn previously.

#### Take out the impact of the pandemic.

The onset of the global COVID-19 pandemic in 2020 significantly altered the business landscape for numerous companies. Given the situations introduced by the pandemic, this paper opted to exclude the 2020 data from the regression analysis to test the robustness. Using ESG_*d* as the independent variable, the regression results (Table 4) show that the coefficient of ESG_*d* is 0.111 at the 1% significance level, indicating that ESG is significantly positively correlated with corporate performance, consistent with the conclusions drawn above.

### Endogeneity test

To test whether there is endogeneity in the model, the following endogeneity tests were conducted.

#### Lagged independent variables.

The benchmark results in Table 3 show that good ESG performance can significantly improve corporate performance. However, firms with good performance may themselves place more emphasis on ESG investment, potentially causing a reverse causality problem. To address this endogeneity issue, the independent variable (*ESG*) is lagged by one period (*L.ESG*) and two periods (*L2.ESG*) for regression analysis. The regression results are shown in Table 5. At the 5% significance level, the lagged one-period and two-period regression coefficients are 0.098 and 0.082, respectively. The results indicate that after considering the reverse causality issue, ESG performance is still significantly positively correlated with corporate performance. The results remain robust, supporting hypothesis H1.

**Table 3:** Benchmark regression results.

Variable	(1)	(2)	(3)	(4)	(5)
***TurbinQ***	***TurbinQ***	***TurbinQ***	***TurbinQ***	***TurbinQ***
*ESG*	0.092^***^	0.114^***^			
	(3.17)	(4.08)			
*E*			0.024		
			(1.12)		
*S*				0.108^***^	
				(3.93)	
*G*					0.130^***^
					(5.75)
*FixedRatio*	-1.102^***^	-1.612^***^	-1.632^***^	-1.613^***^	-1.658^***^
	(-5.43)	(-7.26)	(-7.28)	(-7.25)	(-7.46)
*Size*	-0.647^***^	-0.556^***^	-0.544^***^	-0.556^***^	-0.544^***^
	(-19.38)	(-16.75)	(-16.16)	(-16.68)	(-16.40)
*Age*	-0.524^***^	-0.129	-0.140	-0.129	-0.164
	(-4.29)	(-0.96)	(-1.03)	(-0.97)	(-1.23)
*Top*	0.009^***^	0.009^***^	0.009^***^	0.009^***^	0.008^***^
	(3.67)	(3.86)	(3.94)	(3.87)	(3.43)
*Growth*	0.475^***^	0.513^***^	0.500^***^	0.510^***^	0.522^***^
	(7.17)	(8.41)	(8.18)	(8.36)	(8.53)
*Constant*	18.196^***^	14.954^***^	15.139^***^	14.959^***^	14.644^***^
	(25.35)	(19.12)	(19.25)	(19.11)	(18.79)
Industry	No	Yes	Yes	Yes	Yes
Year	No	Yes	Yes	Yes	Yes
Observations	5,666	5,666	5,666	5,666	5,666
Adj R^2^	0.237	0.343	0.339	0.343	0.349

Note: t-values are shown in parentheses, and results are firm-level clustered;

* ,

** , and

*** indicate significance at the 10%, 5%, and 1% levels, respectively.

**Table 5:** Regression with lagged independent variables.

Variable	(1)	(2)
	*TurbinQ*	*TurbinQ*
*L.ESG*	0.098^***^	
	(3.06)	
*L2.ESG*		0.082^**^
		(2.45)
*FixedRatio*	-1.411^***^	-1.457^***^
	(-5.71)	(-5.44)
*Size*	-0.400^***^	-0.297^***^
	(-10.74)	(-7.53)
*Age*	-0.125	-0.185
	(-0.83)	(-1.09)
*Top*	0.007^***^	0.006^**^
	(2.59)	(2.23)
*Growth*	0.452^***^	0.465^***^
	(6.54)	(6.09)
*Constant*	11.289^***^	9.113^***^
	(12.70)	(9.36)
Industry	Yes	Yes
Year	Yes	Yes
Observations	4,079	3,213
Adj R^2^	0.222	0.168

Note: t-values are shown in parentheses, and results are firm-level clustered;

* ,

** , and

*** indicate significance at the 10%, 5%, and 1% levels, respectively.

#### Instrumental variable method.

To further address the endogeneity issue in the model, this paper constructs an instrumental variable. In previous studies, industry or regional average ESG performance has often been used as an instrumental variable for corporate ESG ratings [41,95,96]. However, this method may not satisfy the exclusivity principle [97]. In this study, due to the impact of industry characteristics and regional economic development, corporate performance often exhibits high industry heterogeneity and regional differences. Industry or regional average ESG performance may directly influence a company’s ESG rating and may also directly affect corporate performance without going through the ESG rating. Therefore, this paper refers to the method of Zhong [98], using ESG fund holdings data as the instrumental variable for corporate ESG performance. The reasons are as follows:

Firstly, as significant institutional investors, ESG funds can influence corporate operations through mechanisms such as “voting with their feet” [99–101]. ESG-themed funds can also enhance the ESG levels of listed companies, thereby satisfying the relevance principle. Secondly, ESG funds are unlikely to directly affect the performance development of listed companies. This is because ESG funds seldom directly engage with the operations of listed companies. Instead, they improve ESG performance by actively communicating with company management. The fund holdings and changes are primarily dependent on the decisions of fund managers [102]. Additionally, corporate performance largely depends on the company’s own operational development, information that is challenging for fund investment professionals to predict, thus meeting the exclusivity requirement. Overall, ESG fund holdings can be viewed as an exogenous variable, as their investment decisions are often based on ESG scores and sustainability criteria, which are relatively independent of a firm’s financial decisions. And there is a significant positive correlation between ESG fund holdings and ESG performance of enterprises. Therefore, ESG fund holdings satisfy the property of instrumental variables.

The first stage model of the instrumental variable regression is:

${ESG}_{i,t}=\beta_{0}+\beta_{1}{Fund}_{i,t}+\beta_{2}{Control}_{i,t}+{Industry}_{i}+{Year}_{t}+\varepsilon_{i,t}$ (2)

where ${ESG}_{i,t}$ represents the ESG rating of firm *i* in year *t*, ${Fund}_{i,t}$ represents the number of ESG funds holding the s*t*ock of firm *i* in year *t*, and the remaining variables are the same as in model (1). In the second stage of the instrumental variable regression, *t*he fitted values from the ESG rating regression are used as the dependent variable to estimate model (1). Since the influence of mutual funds has a certain lag, the number of funds is lagged by one period in the regression process. Table 6 presents the two-stage regression results of the instrumental variable approach. Column (1) of Table 6 shows the first-stage regression results, where the coefficient of *L.Fund* is significantly positive, reflecting the positive correlation between the instrumental variable (*Fund*) and the ESG rating. The p-value for F statistic is 0.048, which is significant at 1% level. The under-identification statistic and weak identification statistic indicate that there are no issues with weak instruments or under-identification. Column (2) of Table 6 presents the second-stage regression results. The p-value for F statistic is 0.000, which is significant at 1% level. It aligns with the benchmark regression results, demonstrating that an improvement in ESG rating can significantly enhance corporate performance.

**Table 6:** Instrumental variable regression.

Variable	(1)	(2)
	*ESG*	*TurbinQ*
*ESG*		2.717^***^
		(0.401)
*L.Fund*	0.187^***^	
	(0.028)	
*Control*	Yes	Yes
Industry	Yes	Yes
Year	Yes	Yes
F Statistic	8.01	7.54
Under-identification Statistic	11.36	
Weak-identification Statistic	29.46	
Observations	4,070	4,070

Note: t-values are shown in parentheses, and results are firm-level clustered;

* ,

** , and

*** indicate significance at the 10%, 5%, and 1% levels, respectively.

## Heterogeneity analysis

### Heterogeneity in ownership structure

The ownership structure of firms may affect the relationship between ESG performance and corporate performance [39,41]. Ownership structures can be categorized into state-owned enterprises (SOEs) and non-state-owned enterprises (non-SOEs). As the demand for sustainable development becomes increasingly important, SOEs have become a key mechanism through which the government promotes high-quality development. According to the research by Deng and Li [103], compared to non-SOEs, SOEs may face stricter environmental regulations and market supervision. Consequently, SOEs are required to undertake greater social responsibilities and are more committed to improving their ESG levels. Additionally, as a vital force in national economic development, SOEs can leverage improvements in ESG performance to enhance both their economic and social benefits, thereby boosting their overall development performance.

Table 7 presents the regression results of ownership structure heterogeneity. The inter-group coefficient test shows that there is a significant difference between state-owned enterprises (SOEs) and non-state-owned enterprises (non-SOEs) at the 10% level. At the 1% level, the *ESG* coefficient for SOEs is 0.146, while for non-SOEs it is 0.086, with the coefficient for SOEs being significantly higher than that for non-SOEs. Therefore, it is believed that ESG has a more significant positive impact on the performance of SOEs. A possible explanation is that due to their state-owned nature, SOEs are subject to social and national supervision. When SOEs actively assume environmental and social responsibilities, their sustainable operational capabilities receive better feedback, generating more positive social responses, and thus enhancing their own performance.

**Table 7:** Regression results of ownership structure heterogeneity.

Variable	SOEs	Non-SOEs
	(1)	(2)
	*TurbinQ*	*TurbinQ*
*ESG*	0.146^***^	0.086^***^
	(2.89)	(2.64)
*Fixed*	-1.915^***^	-0.925^***^
	(-5.55)	(-3.08)
*Size*	-0.503^***^	-0.594^***^
	(-8.87)	(-14.25)
*Age*	-0.117	-0.071
	(-0.39)	(-0.46)
*Top*	-0.004	0.016^***^
	(-1.11)	(5.08)
*Growth*	0.413^***^	0.509^***^
	(3.87)	(7.23)
*Constant*	13.995^***^	15.443^***^
	(10.58)	(14.89)
Industry	Yes	Yes
Year	Yes	Yes
Observations	1,764	3,902
Adj R^2^	0.305	0.361

Note: t-values are shown in parentheses, and results are firm-level clustered;

* ,

** , and

*** indicate significance at the 10%, 5%, and 1% levels, respectively.

### Heterogeneity in industry nature

The nature of the industry may influence the relationship between ESG performance and corporate performance [104]. Industry nature can be categorized into manufacturing and non-manufacturing. Due to significant differences in production operations and business models between manufacturing and non-manufacturing sectors, their attention to ESG performance, the cost of investment, and the impact on corporate performance may also differ. The manufacturing sector, due to its production needs, includes many heavily polluting industries and should assume greater social responsibility in the process of improving the environment. If this issue is addressed, the ESG rating of these companies will also improve, leading to better corporate performance.

Table 8 displays the regression results for industry nature heterogeneity. The inter-group coefficient test reveals a significant difference between the manufacturing and non-manufacturing sectors at the 1% level. At the 5% level, the coefficient for manufacturing is 0.181, while for non-manufacturing it is 0.065, with the coefficient for manufacturing being significantly higher than for non-manufacturing. Thus, it is believed that ESG has a more significant positive impact on the performance of the manufacturing sector. A possible explanation is that the manufacturing sector includes many heavily polluting industries. If these companies improve their ESG performance, the positive signals can be transmitted to the external environment, making them more likely to attract social attention, thereby enhancing their social and economic benefits and improving corporate performance.

**Table 8:** Regression results of industry nature heterogeneity.

Variable	Manufacturing	Non-Manufacturing
	(1)	(2)
	*TurbinQ*	*TurbinQ*
*ESG*	0.181^***^	0.065^**^
	(3.68)	(1.98)
*Fixed*	-1.760^***^	-1.380^***^
	(-4.71)	(-5.01)
*Size*	-0.572^***^	-0.533^***^
	(-9.75)	(-13.96)
*Age*	0.246	-0.372^**^
	(1.14)	(-2.30)
*Top*	0.018^***^	0.004
	(4.27)	(1.56)
*Growth*	0.544^***^	0.488^***^
	(5.16)	(6.63)
*Constant*	13.740^***^	15.425^***^
	(9.62)	(17.45)
Industry	Yes	Yes
Year	Yes	Yes
Observations	2,255	3,411
Adj R^2^	0.305	0.375
Group Coefficient Test	0.001	

Note: t-values are shown in parentheses, and results are firm-level clustered;

* ,

** , and

*** indicate significance at the 10%, 5%, and 1% levels, respectively.

### Moderating effect

As the primary advocate and regulator of ESG ratings, the government’s influence on corporate development has grown significantly in recent years. The impact of ESG ratings on corporate performance may vary under different levels of government subsidies. Understanding the moderating effect will help the government provide targeted subsidies to different enterprises. Therefore, this study investigates the moderating effect of government subsidies on the relationship between ESG ratings and corporate performance. To avoid multicollinearity caused by interaction terms and to prevent biased parameter estimates, the interaction terms in the model are mean-centered. Table 9 presents the regression results of the moderating effect.

**Table 9:** Moderating effect regression.

Variable	(1)	(2)	(3)
	*TurbinQ*	*TurbinQ*	*TurbinQ*
*ESG*	0.114^***^		0.109^***^
	(4.08)		(3.90)
*Govern*		0.045^**^	0.037*
		(2.13)	(1.72)
ESG*Govern			0.043^***^
			(3.52)
*FixedRatio*	-1.612^***^	-1.590^***^	-1.556^***^
	(-7.26)	(-7.11)	(-7.05)
*Size*	-0.556^***^	-0.575^***^	-0.597^***^
	(-16.75)	(-15.15)	(-16.09)
*Age*	-0.129	-0.128	-0.115
	(-0.96)	(-0.95)	(-0.87)
*Top*	0.009^***^	0.009^***^	0.009^***^
	(3.86)	(3.86)	(3.91)
*Growth*	0.513^***^	0.500^***^	0.513^***^
	(8.41)	(8.11)	(8.40)
*Constant*	14.954^***^	15.107^***^	16.283^***^
	(19.12)	(19.15)	(18.76)
Industry	Yes	Yes	Yes
Year	Yes	Yes	Yes
Observations	5,666	5,666	5,666
Adj R^2^	0.343	0.340	0.347

Note: t-values are shown in parentheses, and results are firm-level clustered;

* ,

** , and

*** indicate significance at the 10%, 5%, and 1% levels, respectively.

Column (3) shows that at the 1% level, the interaction term (ESG*Govern) is significantly positive, with a coefficient of 0.043, and the independent variable (*ESG*) is significantly positive, with a coefficient of 0.109. The results indicate that government subsidies significantly enhance the impact of ESG ratings on corporate performance, acting as a positive moderator. The possible explanations are as follows: Government subsidies, as an incentive policy provided by the government, promote continuous internal governance improvement and environmental protection through financial support and directional guidance, thereby improving corporate ESG performance. Zhong [105] suggest that companies receiving government subsidies have more resources and capabilities to undertake environmental and social responsibilities. To continue receiving government subsidies and maintain good government-business relations, companies will consistently improve their ESG performance. Meanwhile, under the incentive of government subsidies, companies will continuously enhance their performance to obtain more government financial support. Furthermore, government subsidies have a strong guiding effect, making companies that receive them more likely to gain recognition from external investors, thereby accessing more financing channels and improving corporate performance. H2 is thus verified.

### Mechanism analysis

In the market, investors face an information disadvantage and may lack a complete understanding of the enterprises. Analysts, serving as market information intermediaries, can excavate and analyze corporate information, predict future market prospects, and provide investment advice and assistance to investors, playing a vital role in market information integration. Good ESG performance by a company demonstrates its proactive assumption of social responsibility, making it easier to gain investor trust and attract more analyst coverage [106,107]. Drawing on the research on mechanisms by Hou [108], this paper further conducts a mechanism analysis of how corporate ESG performance affects corporate performance. The study draws on Dell [109] and Chen et al. [110] methods to demonstrate mediating effects. The model 3 constructed is as follows:

${Analyst}_{i,t}\;=\;\alpha_{0}+\alpha_{1}{ESG}_{i,t\;}+{\;\alpha }_{2}{Control}_{i,t}+{Firm}_{i}+{Year}_{t}\;+\varepsilon_{i,t}$ (3)

where ${ESG}_{i,t}$ represents the ESG rating of firm *i* in year *t*, ${Analyst}_{i,t}$ represents the analyst coverage for firm *i* in year *t*, ${Control}_{i,t}$ represents control variables, and $\varepsilon_{i,t}$ is the random disturbance term. The model controls for industry (${Industry}_{i}$) and *t*ime (${Year}_{t}$) effects. The results are shown in Table 10. Column (1) shows that at the 1% level, ESG performance is significantly positive, with a coefficient of 0.143, indicating that an improvement in ESG rating can significantly increase analyst coverage. When front-loading analyst coverage by one period (*F.Analyst*), the results remain unchanged. H3 is thus verified.

**Table 10:** Mechanism regression.

Variable	(1)	(2)
	*Analyst*	*F.Analyst*
*ESG*	0.143^***^	0.121^***^
	(9.17)	(6.48)
*FixedRatio*	-0.686^***^	-0.558^***^
	(-4.98)	(-3.56)
*Size*	0.315^***^	0.279^***^
	(18.23)	(13.61)
*Age*	-0.338^***^	-0.329^***^
	(-4.38)	(-3.67)
*Top*	-0.001	-0.001
	(-0.38)	(-0.31)
*Growth*	0.169^***^	0.219^***^
	(5.44)	(5.68)
*Constant*	-4.610^***^	-3.667^***^
	(-10.57)	(-7.18)
Industry	Yes	Yes
Year	Yes	Yes
Observations	5,666	5,666
Adj R^2^	0.205	0.160

Note: t-values are shown in parentheses, and results are firm-level clustered;

* ,

** , and

*** indicate significance at the 10%, 5%, and 1% levels, respectively.

Simultaneously, increased analyst coverage can also enhance corporate performance. Higher analyst coverage indicates greater growth potential for the company, making it more likely to achieve higher performance in the future [111]. As information intermediaries, analysts capture signals from companies in the market, reducing information asymmetry between transaction parties, thereby attracting potential investors and improving financing efficiency and expanding financing channels [106,112]. Additionally, analysts act as market regulators, overseeing corporate activities, detecting fraudulent behavior, and alerting external investors [113,114]. Consequently, numerous studies suggest that higher analyst coverage helps enhance corporate performance. Therefore, analyst coverage constitutes a key mechanism through which corporate ESG performance influences corporate performance; improved ESG performance attracts analyst coverage, which in turn boosts corporate performance.

## Discussion

### Research findings and theoretical implications

This study provides comprehensive empirical evidence on the relationship between corporate ESG performance and financial outcomes in Chinese A-share listed companies. The findings reveal that improved ESG performance significantly enhances corporate financial performance, particularly in state-owned enterprises (SOEs) and the manufacturing sector. This positive relationship demonstrates that companies’ commitment to environmental, social, and governance responsibilities can create tangible financial value. The stronger effect observed in SOEs can be attributed to their unique position in China’s economy and their role in implementing national sustainability policies. Similarly, the more pronounced impact in the manufacturing sector underscores the importance of ESG practices in industries with significant environmental footprints.

The study also finds that government subsidies play a crucial moderating role, amplifying the positive impact of ESG performance on financial outcomes. This finding highlights the effectiveness of policy support in promoting sustainable business practices and suggests that financial incentives can create a virtuous cycle of sustainable development and improved financial performance. Furthermore, analyst coverage serves as a key mechanism linking ESG performance to financial outcomes, suggesting that market intermediaries play a crucial role in translating ESG information into market value.

These findings can be interpreted through several theoretical frameworks. From the perspective of stakeholder theory, the positive ESG-performance relationship demonstrates how effective stakeholder management through ESG practices can enhance firm value. Companies with strong ESG performance better satisfy stakeholder expectations, leading to improved financial outcomes through enhanced reputation, customer loyalty, and employee satisfaction. The results also support signaling theory, showing how strong ESG performance signals responsible management and sustainable business practices to market participants. This signaling effect is particularly evident in the relationship between ESG performance and analyst coverage. Additionally, the findings, especially regarding the moderating role of government subsidies, align with resource dependence theory. Companies with better ESG performance attract more resources, including government support and analyst attention, which enhances their competitive advantage.

### Comparison with previous literature

The results of this research both support and extend existing literature in several important ways. The positive relationship between ESG performance and financial outcomes aligns with previous studies by Alareeni and Hamdan [57] and Al Hawaj and Buallay [58], who found similar positive associations in different market contexts. However, this study provides unique insights into the Chinese market context, particularly regarding the role of state-owned enterprises in ESG implementation.

The identification of government subsidies as a significant moderating factor adds a new dimension to the existing literature on the role of government in promoting corporate sustainability. While previous studies have examined the direct effects of government policies on ESG performance, this research highlights how government financial support can amplify the financial benefits of strong ESG performance.

The finding on the role of analyst coverage as a mechanism linking ESG performance to financial outcomes contributes to the growing literature on the information intermediary role of financial analysts in ESG contexts. It provides empirical evidence supporting the work of Amel-Zadeh and Serafeim [107], demonstrating how ESG performance influences investor perceptions and decision-making through enhanced information dissemination and scrutiny by analysts.

### Research limitations

This study, despite its contributions, has several important limitations that warrant acknowledgment. The focus on A-share listed companies, while providing valuable insights into the Chinese market, may limit the generalizability of findings to other market contexts. The sample selection process, driven by data availability requirements, could introduce potential biases that affect the interpretation of results.

Methodological limitations also exist in the research approach. The reliance on CSI ESG ratings, while providing a standardized measure of ESG performance, may not capture all relevant aspects of corporate sustainability practices. Despite attempts to address endogeneity through instrumental variables, concerns about causal inference remain. The potential for omitted variable bias, despite the inclusion of numerous control variables, cannot be completely eliminated.

The study period from 2015 to 2022, while providing recent evidence, may not fully capture the long-term effects of ESG initiatives. The results might be influenced by specific economic conditions and policy environments during this period, potentially affecting their generalizability across different time frames. These limitations suggest opportunities for future research to address these constraints through alternative methodological approaches and expanded data sets.

### Practical implications and policy recommendations

#### For companies.

Companies should actively assume social responsibility and enhance their ESG performance through systematic and measurable approaches. In recent years, as the demand for sustainable development has gained increasing attention, corporate ESG performance has gradually become a key focus for investors. Research indicates that strong ESG performance can send positive signals to the external environment, demonstrating a company’s proactive fulfillment of social responsibility, making it more likely to attract stakeholder attention, gain social support and financing, and thereby enhance its performance and operational capabilities [30,115,116]. To achieve this goal, companies should establish dedicated ESG management systems, develop comprehensive disclosure frameworks, and integrate ESG considerations into their strategic planning. For state-owned enterprises, setting industry-leading ESG standards and developing innovative green practices is crucial, given their significant role in implementing national sustainability policies [117]. For manufacturing enterprises, given their high proportion of heavily polluting industries, they should focus on investing in clean technology, implementing energy efficiency programs, and developing sustainable supply chain management systems to enhance ESG performance and improve corporate performance [118].

#### For government and regulators.

The government should strengthen both policy support and regulatory oversight of corporate ESG performance through a differentiated approach. Government subsidies should be designed with clear criteria and performance metrics, providing targeted financial support to companies based on their ESG achievements and industry characteristics. Such performance-based subsidies can provide both financial support and market confidence to companies, enabling them to better fulfill their environmental responsibilities [119]. Additionally, government subsidies have a guiding effect, attracting social investors to focus more on companies, expanding their financing channels, and promoting corporate performance. To enhance policy effectiveness, regulatory authorities should establish a standardized ESG evaluation system and increase policy support and financial guarantees for companies with good ESG performance [120]. Furthermore, since our findings indicate that the positive impact of ESG performance on corporate performance is more significant in state-owned enterprises, ESG metrics should be incorporated into their assessment systems with appropriate incentive mechanisms. For the manufacturing sector, particularly heavily polluting industries, authorities should develop specialized support programs that link financial incentives to specific environmental improvements and green transformation targets [121].

#### For market participants.

The role of analysts in market information transmission and supervision should be emphasized, particularly in emerging markets where information asymmetry is typically high. Good corporate ESG performance indicates proactive social responsibility and sustainable development capabilities, which can attract more analyst coverage. As information intermediaries in the market, analysts can convey corporate information to investors, reducing the degree of information asymmetry between companies and investors, providing investment advice, and expanding financing channels for companies. Additionally, analysts can supervise corporate operations; companies under analyst scrutiny are more likely to regulate their behavior, actively assume social responsibility, and focus on improving their operational capabilities. To enhance this market mechanism, companies should establish standardized ESG information disclosure channels with analysts and develop regular communication protocols [122]. Regulatory authorities should improve external governance mechanisms involving analysts and other entities, including developing clear guidelines for ESG analysis and reporting standards. This would help fully leverage the supervisory role of analysts in promoting efficient and coordinated market operations.

### Future research directions

Future research should expand the geographical scope to include international comparisons, examining how ESG effects vary across different institutional contexts and market environments. This could include comparative studies of ESG practices in other emerging markets and investigations of cultural factors affecting ESG implementation.

Methodological advances should focus on developing more sophisticated analytical approaches, including the application of advanced econometric techniques and more comprehensive ESG measurement methods. There is also a need for studies examining non-linear relationships and interaction effects between different ESG components.

Long-term studies are needed to better understand the temporal dynamics of ESG implementation and its effects. This includes analysis of ESG impacts across different economic cycles and investigation of the processes through which ESG initiatives are implemented and their effects realized. Such research would contribute to both academic knowledge and practical understanding of sustainable business practices.

The exploration of additional mechanisms linking ESG performance to financial outcomes represents another important avenue for future research. This includes investigating how different stakeholder groups respond to ESG initiatives and how these responses collectively influence corporate performance. Such investigations would enhance understanding of the pathways through which ESG practices create value for organizations and society.

## Conclusions

This study examines the relationship between corporate ESG performance and financial outcomes using data from Chinese A-share listed companies from 2015 to 2022. The empirical analysis yields several significant findings. First, ESG performance exhibits a positive relationship with corporate financial performance, with this effect being particularly pronounced in state-owned enterprises and the manufacturing sector. Second, government subsidies play a substantial moderating role in this relationship, amplifying the positive impact of ESG initiatives on corporate performance. Third, the research identifies analyst coverage as a key mechanism through which ESG performance enhances corporate performance.

These findings make several important contributions to the literature. The study provides empirical evidence from the Chinese market context, offering insights into how ESG practices influence firm performance in an emerging market setting. The identification of government subsidies as a moderating factor advances understanding of how policy support can enhance the effectiveness of ESG initiatives. Furthermore, the research clarifies the role of market intermediaries in translating ESG performance into financial outcomes through analyst coverage.

From a practical perspective, these findings have significant implications for corporate strategy and policy development. Companies, particularly those in the manufacturing sector and state-owned enterprises, should recognize the value-creating potential of strong ESG performance. Policy makers should consider the effectiveness of financial incentives in promoting sustainable business practices. Market participants, including investors and analysts, should incorporate ESG factors more systematically into their evaluation processes.

The research suggests that the path toward sustainable development in China’s corporate sector requires coordinated efforts from multiple stakeholders. As ESG considerations continue to gain prominence in global markets, the findings of this study provide valuable guidance for businesses seeking to balance environmental and social responsibilities with financial performance. Future research could further explore these relationships across different market contexts and time periods, contributing to a more comprehensive understanding of how ESG practices create value in an evolving business environment.

## Supporting information

S1 File ROAA data. (XLSX)
